# Use of unconventional antithyroid therapy in patients with thiamazole agranulocytosis in the context of the COVID-19 pandemic

**DOI:** 10.20945/2359-3997000000453

**Published:** 2022-03-08

**Authors:** Julio César Alvarez-Gamero, Victor Raúl García-Ruiz, Jose Luis Paz-Ibarra

**Affiliations:** 1 Edgardo Rebagliati Martins National Hospital Peruvian University Cayetano Heredia Endocrinology Service Lima Peru Endocrinology Service, Edgardo Rebagliati Martins National Hospital Peruvian University Cayetano Heredia, Lima, Peru; 2 Edgardo Rebagliati Martins National Hospital National University of San Marcos Endocrinology Service Lima Peru Endocrinology Service, Edgardo Rebagliati Martins National Hospital National University of San Marcos, Lima, Peru; 3 Hospital Nacional Edgardo Rebagliati Martins National University of San Marcos Endocrinology Service Lima Peru Endocrinology Service, Hospital Nacional Edgardo Rebagliati Martins National University of San Marcos, Lima, Peru

**DEAR EDITOR,**

We have read with interest the article published by Martins JRM, which that tells us about the side effects of anti-thyroid medications in hyperthyroidism and the possibility of definitive treatment once this has occurred ( 1 ). The SARS-CoV-2 virus pandemic has displaced much of the health system's resources, compromising care for other diseases, including hyperthyroidism. Various documents have been presented with the intention of outlining its treatment during the pandemic, focusing it on the use of oral antithyroid therapy due to the impossibility of performing thyroidectomies or administration radioiodine (131I). However, the course to follow in any adverse effect of these has not been clearly indicated, which is worrying, given the lack of alternatives in this context.

Agranulocytosis is an infrequent (0.2%) but serious adverse effect of thiamazole ( 2 ). Alternative treatment with 131I or surgery is effective; however, it was not available in the first months due to a state of emergency in our country (Peru) due to SARS-CoV-2. In this scenario, the use of unconventional antithyroid therapy is configured as an option: compounds containing iodine, lithium carbonate, glucocorticoids, cholestyramine; in which plasmapheresis could also be considered ( 2 ).

Lithium is actively concentrated in thyroid follicular cells, 3-4 times more than in plasma, which main mechanism of action is decrease in the release of thyroid hormones through the inhibition of colloidal pinocytosis ( 3 ). In a study of 13 patients, with severe adverse reactions to conventional antithyroid therapy or treatment failure, 8 patients responded to lithium carbonate (response defined as a decrease in free T4 > 50% with clinical improvement) 2 weeks after treatment, while 4 patients responded within 3 to 5 weeks later, with a mean dose of 750 mg/d ( 4 ). In another study of 51 patients with liver injury or leukopenia secondary to the use of thionamides, twelve (23.5%) obtained clinical remission after one year of follow-up, 6 patients (11.8%) relapsed after withdrawal, the dose used was 500-750 mg/d ( 5 ).

The American Thyroid Association does not recommend plasmapheresis as a preoperative preparation in the definitive treatment of hyperthyroidism ( 2 ). The American Society of Apheresis recommends its use in case of thyroid storm as second-line therapy, but doesn't make any recommendation in patients with hyperthyroidism without a thyroid storm ( 6 ); however, it could be used in patients with significant adverse effects from thionamides in whom an effective and rapid reduction of thyroid hormone levels is required.

In conclusion, although the health emergency has brought many setbacks, it is also an opportunity to direct attention to the use of alternative treatments that, in situations like this, can be useful.
